# Sudden Death in a Patient with Carney's Complex

**DOI:** 10.5812/aapm.9111

**Published:** 2013-03-26

**Authors:** James Adam Rothschild, Melissa Kreso, Martin Slodzinski

**Affiliations:** 1 Department of Anesthesiology and Critical Care Medicine, Johns Hopkins School of Medicine, Baltimore, Maryland, USA; 2 Department of Anesthesiology, University of Rochester Medical Center, Rochester, USA

**Keywords:** Carney Complex, Myxoma, Death, Sudden, PRKAR1A Protein, Human

## Abstract

Carney’s complex is a rare autosomal dominantly inherited multiple endocrine neoplasia syndrome that involves spotty skin pigmentations, recurrent cardiac myxomas, endocrine hyperactivity, pituitary adenomas, peripheral nerve tumors, testicular tumors, and ovarian lesions. We present a case of sudden cardiac death in a 40 year old female with a history of Carney’s complex with recurrent cardiac myxomas presenting for exploratory laparotomy and enblock adnexal resection of a slowly enlarging right sided ovarian mass. This case highlights the risk for sudden death in these patients as well as the preoperative assessment that should be undertaken by the anesthesiologist as it relates to Carney’s complex.

## 1. Introduction

Carney’s complex is a rare autosomal dominantly inherited multiple endocrine neoplasia syndrome that involves spotty skin pigmentations, cardiac myxomas, endocrine hyperactivity, pituitary adenomas, peripheral nerve tumors, testicular tumors, and possibly ovarian lesions (1). Prior to its discovery in 1975 by JA Carney, these symptom clusters were referred to as NAME (Nevi, Atrial Myxoma, Myxoid neurofibroma, and Ephelides) as well as LAMB (Lentigines, Atrial Myxoma, and Blue nevi) syndromes (2). Despite the propensity to develop cardiac myxomas, Carney complex has rarely been associated with sudden and near death events. We present a case of sudden death in a patient with a history of Carney’s complex.

## 2. Case Report

The patient was a 40 year old caucasian woman (102kg, 173cm) who presented for exploratory laparotomy and enblock resection of adnexa and surrounding peritoneum for a slowly enlarging 12cm x 10cm right sided adnexal mass, followed for over the course of two years on serial pelvic ultrasounds. Her medical history was significant for a diagnosis of Carney’s complex, alcohol related cardiomyopathy, congestive heart failure, complete heart block (pacemaker dependent), aortic stenosis (valve gradient of 72 mmHg), moderate pulmonary hypertension (RVSP of 45 mm Hg), mitral regurgitation, avascular necrosis of the hip, and ovarian cysts. There was no evidence of Cushing’s syndrome. Her surgical history included two open heart procedures for excision of cardiac myxomas (left atrial in 1985, and right atrial and right ventricle in 1990), aortic and mitral valve replacement (1985), pacemaker placement for complete heart block (1985,1990), and a vaginal hysterectomy (1992). Originally diagnosed with Carney’s complex at the age of 20, the patient remained medically stable and in her usual state of health until 1997 when she presented to the emergency department with increasing shortness of breath, lower extremity edema and palpitations. Cardiac catheterization and transesophageal echocardiogram (TEE) at that time revealed no coronary artery disease, elevated right sided filling pressures, moderate global LV dysfunction, moderate to severe mitral regurgitation and a fixed posterior mitral valve leaflet. Ejection Fraction at that time was 30% on TEE. Thoracic computerized tomogram revealed a recurrence of the right ventricular myxoma (1.5 cm). Cardiothoracic surgery was consulted and recommended aggressive medical management including aggressive diuresis, ACE inhibition, and anticoagulation. The patient was subsequently discharged after a two week hospitalization. Three years later (2000), the patient began to experience pelvic discomfort, worsening exercise tolerance, arthralgias, fatigue, photosensitivity and occasional night sweats. Physical examination and ultrasound imaging revealed a 12 x 10 cm freely mobile mass without nodularity in the right pelvic cul-de-sac. Pulmonary, abdominal, and extremity exams were otherwise unremarkable. Rheumatoid factor, erythrocyte sedimentation rate, Lyme titer, TSH, ferrittin, CBC, electolytes and CA-125 were normal. Electrocardiogram revealed a paced rhythm. Echocardiogram performed 3 months prior revealed a dilated left atrium and ventricle, normal LV systolic function, thickened mitral valve, moderate mitral and tricuspid regurgitation and moderate to severe aortic stenosis. Ejection fraction at that time was 60%. Of note, the patient had discontinued use of alcohol three year prior following the hospitalization mentioned above. The decision was made to undergo elective excision of the adnexal mass. After application of standard ASA monitors and insertion of a radial arterial line, the patient was preoxygenated and induced with 14 mg of etomidate, 100 mg of lidocaine, 250 mcg of fentanyl and 80mg of rocuronium for tracheal intubation. General anesthesia was maintained with isoflurane and a total of 550 mcg of fentanyl. A TEE probe was placed without complication and echocardiogram revealed, a right atrial mass extending into the SVC, a dilated left atrium, moderate MVP, MR and TR and a normal LV. The surgeons performed an extensive lysis of adhesions as well as an exploratory laparotomy, appendectomy, and bilateral salpingo-oophorectomy with resection of surrounding peritoneum. Frozen section intraoperatively revealed endometriosis in the right ovary without pelvic involvement. The patient remained hemodynamically stable throughout the procedure. Neuromuscular blockade was reversed and the patient was extubated in the operating room and transported to the surgical intensive care unit (SICU), awake and comfortable. Estimate blood loss was 150cc with 4 liters of crystalloid resuscitation during the three hour case. Postoperative hemoglobin was 12.1. The patient remained in the SICU for two days postoperatively for fluid management secondary to postoperative volume overload. She was aggressively diuresed and started on enoxaparin, which was converted to warfarin on post-operative day (3). She tolerated diuresis well and was transferred to the surgical floor on post-operative day (3). On that day she had one temperature recording of 38.2 with a mildly elevated white blood cell count (WBC) of 11500. On subsequent measurement she was found to be afebrile with normal WBC and did not require post-operative antibiotics. On postoperative day (4) the patient was afebrile, all labs were stable and final hemoglobin was 10.9. The patient had an otherwise uneventful hospital stay and was discharged to home on postoperative day (4) on her original PO medication regimen. Unfortunately, four hours after arriving home, the patient became febrile and within minutes became unresponsive. Cardiopulmonary resuscitative attempts were unsuccessful and the patient was pronounced dead on the day of hospital discharge. An autopsy was performed 60 hours postmortem. External examination revealed multiple pigmented macules over most of the body with no cushingoid or acromegalic features. Cardiac examination revealed dense adhesions throughout the mediastinum as well as pericardial adhesions to the epicardium. RA and RV were dilated and a sessile red/tan1.5 cm mass was present in the RV. The St. Jude’s aortic valve was intact; however, the infravalvular outflow tract was markedly narrowed, consistent with the history of severe aortic stenosis. There was no evidence of coronary artery disease and only patchy fatty streaks were found within the aorta. The pulmonary exam revealed edematous lungs but no evidence of thromboemboli or consolidation. The liver and spleen were enlarged with evidence of chronic hepatic congestion. Examination of the adrenals revealed several small black nodules in the adrenal cortex.

## 3. Discussion

Carney’s complex is one of the most recently discovered multiple endocrine neoplastic syndromes. It is a relatively rare disease with just over 500 cases having been registered by the NIH/Mayo Clinic and Cochin Center (5). It is more prevalent in males than females and has been reported in a diverse ethnic population. Carney Complex has a strong genetic component (70% familial) and has a median age of diagnosis of 20 years (5, 6). Carney’s complex is diagnosed with the presence of two of the following manifestations: skin lentigenes, cardiac myxomas, neurofibromas, pituitary adenomas and signs of endocrine hyperactivity. Alternatively the diagnosis can be made if one manifestation is present and a first degree relative has been diagnosed with Carney complex. The median age of detection is 20 years; however, the first signs of abnormal skin pigmentations may be present as early as birth. Sixty-seven percent of patients have cardiac tumors and 75% develop various skin pigmentation lesions including blue nevi, café au lait spots, and depigmented lesions (6). Lentigines often develop early in the prepubertal period and typically involve lips, conjunctiva, inner and outer canthi, and vaginal and penile mucosa. These skin manifestations are typically benign (5). In recent years, Carney’s complex has been localized to isolated mutations of the PRKAR1A gene on chromosome 17q22-24. PRKAR1A encodes the regulatory subunit type 1-α of protein kinase A, a key regulator of the cyclic adenosine monophosphate dependent signaling pathway and tumor suppressor whose downstream dysfunction has been linked to 45-80% of cases of Carney’s complex (1, 5, 6). A second locus has also been implicated at chromosome 2p168. Cardiac myxomas in Carney complex account for more than 50% of disease specific mortality (5). As opposed to non-familial cardiac myxomas which present in middle age, myxomas related to Carney’s complex are typically diagnosed before the age of 24 and affect both sexes equally. The majority (75%) of myxomas are found in the left atrium in the region of the limbus of the fossa ovalis. Approximately 15%-20% occur in the right atrium, with the remaining 5-10% being in the other chambers (2). Embolism occurs in up to 40% of patients with myxomas in the form of transient ischemic events and strokes (7). Familial myxomas constitute 10% of all myxomas and are likely to recur after surgical resection (22% recurrence rate). Immunochemistry has revealed positivity in both mesenchymal origin (vimentin) as well as several neuroendocrine markers (S-100 and calretinin) (2). In a study of 51 patients with Carney’s Complex, 96% presented with spotty pigmentation, 30% had cardiac myxomas, 32% had pigmented adrenocorticoid disease, 18% had schwannomas, and 8% presented with acromegaly. Of those 51 patients (4) had an associated sudden death or near death experience. When presented with a patient with Carney’s complex, one must appreciate not only the individual challenges, but also the systemic manifestations of the disease. To the anesthesiologist, cardiac myxomas constitute the most lethal manifestation of the syndrome, and the one that demands a vigilant preoperative workup as well as careful post-operative follow-up with a cardiologist. Myxomas alter valvular function via outflow obstruction and valvular growths, and pose the threat of embolization to the brain and other organs. Despite previous myxoma resection in patients with known Carney Complex, one must discern symptoms of outflow obstruction, which could signify possible recurrence. Additionally, one must also be aware of the propensity of these patients to have adrenocorticoid dysfunction including glucose intolerance and cushingoid features. Preoperative management of the Carney’s complex patient should include an ECG and recent echocardiogram to evaluate for the presence of atrial or ventricular myxomas. In addition, an endocrine evaluation is necessary to assess for adrenocorticoid involvement. Excess cortisol can lead to slow wound healing, hyperglycemia, and hypertension. If signs of acromegaly are present, one should recognize the risk of difficult intubation from excess soft tissue growth. In conclusion, we present a single case of sudden post-operative death in a patient with previously diagnosed Carney’s complex. These patients have a predisposition to postoperative cardiac arrhythmias, outflow tract obstruction, and myocardial infarction as a result of their underlying myxomas.

## References

[1] Lodish MB, Stratakis CA (2010). Rare and unusual endocrine cancer syndromes with mutated genes.. Semin Oncol..

[2] Shetty Roy AN, Radin M, Sarabi D, Shaoulian E (2011). Familial recurrent atrial myxoma: Carney's complex.. Clin Cardiol..

[3] Stratakis CA, Papageorgiou T, Premkumar A, Pack S, Kirschner LS, Taymans SE (2000). Ovarian lesions in Carney complex: clinical genetics and possible predisposition to malignancy.. J Clin Endocrinol Metab..

[4] Stratakis CA, Carney JA, Lin JP, Papanicolaou DA, Karl M, Kastner DL (1996). Carney complex, a familial multiple neoplasia and lentiginosis syndrome. Analysis of 11 kindreds and linkage to the short arm of chromosome 2.. J Clin Invest..

[5] Lodish MB, Stratakis CA (2011). The differential diagnosis of familial lentiginosis syndromes.. Fam Cancer..

[6] Saggini A., Brandi ML (2011). Skin lesions in hereditary endocrine tumor syndromes.. Endocr Pract..

[7] Vaughan CJ, Veugelers M, Basson CT (2001). Tumors and the heart: molecular genetic advances.. Curr Opin Cardiol..

